# Computerized cognitive training in post-treatment hematological cancer survivors: a feasibility study

**DOI:** 10.1186/s40814-021-00778-3

**Published:** 2021-01-30

**Authors:** Samantha J. Mayo, Sean B. Rourke, Eshetu G. Atenafu, Rita Vitorino, Christine Chen, John Kuruvilla

**Affiliations:** 1 Lawrence S. Bloomberg Faculty of Nursing, University of Toronto, 155 College St., Suite 130, Toronto, ON M5T 1P8 Canada; 2 Princess Margaret Cancer Centre, University Health Network, Toronto, ON Canada; 3 Li Ka Shing Knowledge Institute, St. Michael’s Hospital, Toronto, ON Canada; 4 Department of Psychiatry, University of Toronto, Toronto, ON Canada; 5 Department of Biostatistics, Princess Margaret Cancer Centre, University Health Network, Toronto, ON Canada; 6 Sunnybrook Health Sciences Centre, Toronto, ON Canada; 7 Division of Medical Oncology and Hematology, Princess Margaret Cancer Centre, University Health Network, Toronto, ON Canada; 8 Department of Medicine, University of Toronto, Toronto, ON Canada

**Keywords:** Cognitive functioning, Hematological cancer, Cancer-related cognitive impairment, Computerized cognitive training, Brain training, Cognitive rehabilitation

## Abstract

**Background:**

Computerized cognitive training (CCT) programs have shown some effectiveness in alleviating cognitive symptoms in long-term cancer survivors. For patients presenting with cognitive symptoms in the early post-treatment phase, the benefit of CCT is unclear. To assess the possibility of testing the effectiveness of CCT in the early post-treatment period, our aim was to investigate the feasibility of an 8-week home-based, online CCT intervention among patients who have recently completed treatment for hematological malignancy.

**Methods:**

This study was a single-arm, non-blinded, feasibility study. All participants were provided with the CCT intervention for an 8-week period. Feasibility was evaluated based on participant adherence and patient perceptions of the intervention, assessed through responses to an acceptability questionnaire and semi-structured interviews at the end of the intervention period.

**Results:**

The feasibility study included 19 patients who had completed treatment for hematological malignancy at a Canadian tertiary cancer center. Adherence to the CCT intervention was limited, with only one participant meeting the criteria for intervention adherence. At the end of the intervention period, participants characterized the program as easy to follow (92%) and felt well-prepared for how to complete the exercises (100%). In semi-structured interviews, participants highlighted post-treatment barriers to intervention adherence that included symptom burden and competing time demands. Participants also suggested improvements to the intervention that could help maintain adherence despite these barriers, such as fostering a sense of accountability, providing personalized feedback and coaching, and enabling opportunities for peer support.

**Conclusions:**

Participation in CCT can be challenging in the post-treatment period for hematological cancers. Further research on the effectiveness of CCT in this setting may require the implementation of strategies that support participants’ engagement with the intervention in the context of symptoms and competing demands, such as establishing a minimum dose requirement and integrating approaches to help promote and sustain motivation.

## Key messages regarding feasibility

- *What uncertainties existed regarding the feasibility?* Computerized cognitive training programs have shown some effectiveness in alleviating cognitive symptoms in long-term cancer survivors. The feasibility of testing the effectiveness of these programs during the early post-treatment period is unclear.
- *What are the key feasibility findings?* Adherence to early intervention with CCT in the post-treatment period may be limited by barriers, such as symptoms and competing time demands, but may be enhanced with greater motivational support.
- *What are the implications of the feasibility findings for the design of the main study?* Studies of CCT effectiveness in the early post-treatment phase require implementation of strategies that help sustain motivation over time and help balance the intervention with competing concerns, including establishing a minimum dose requirement.

## Background

Cancer-related cognitive impairment, comprising difficulties with short-term memory, processing speed, and complex attention/working memory, has been documented after treatment for a range of cancers, including hematological malignancies. Treatment of hematological malignancies, such as lymphoma and multiple myeloma, comprise a range of systemic treatments that have been associated with cognitive effects, including chemotherapy [1–3] and stem cell transplantation [4]. Meta-analytic findings indicate that 12–89% of patients treated for hematologic malignancies meet the criteria for neurocognitive impairment, depending on how impairment is defined [4]. While most patients are expected to recover over time [4], acute deficits in the first few years after treatment for hematological cancers are associated with limitations in medication management, social functioning, employment status, and overall quality of life [5–12]. The burden is compounded with a lack of guidelines for the CRCI management [7, 13–15]. In a study of 715 hematological cancer survivors, “coping with having a bad memory or lack of focus” was the 2nd most frequently endorsed “high/very high” unmet need [16].

The increasing availability and popularity of computerized cognitive training (CCT) programs have expanded interest in their use as a non-pharmacological intervention for maintaining or improving cognitive functioning [17]. Contemporary programs generally guide participants through a prescribed series of computerized exercises designed to improve performance in targeted cognitive domains that are repeated over a period of weeks to months. The exercises target the improvement of perceptual processing of stimuli, based on the hypothesis that this will facilitate improvements in the higher-level cognitive functions (e.g., memory) that rely on the efficiency with which incoming information is received and processed [18, 19]. In the cancer setting [20–24], CCT has been associated with improvement on objective cognitive tests that measure trained domains of cognitive function, but also show transfer effects through improvement in non-targeted cognitive domains, self-reported perception of cognitive functioning [20, 25], and improvements in activities of daily living [26]. However, limitations in the current body of evidence, including the need for research beyond long-term survivors of solid tumors, remain acknowledged [20, 25]. Additionally, other studies have shown either mixed or little improvements related to CCT and suggest that the benefit may vary based on population, dose, and also motivation to engage in these training programs [17].

An advantage of recent CCT programs is that they can also be completed in the home setting. While early studies required supervised CCT to be conducted in-person [22, 27, 28], home-based CCT interventions may offer greater flexibility and access to cognitive training. Home-based CCT interventions have demonstrated good adherence among long-term survivors of solid tumors, and benefits to objectively measured and subjectively assessed cognitive outcomes have been reported [21, 23, 24, 29]. To our knowledge, home-based CCT has yet to be tested in the hematological cancer setting.

To support the design of future trials, we sought to investigate whether home-based CCT would be feasible among hematological cancer survivors, particularly in the early post-treatment phase where interference of cognitive effects may be high. Notably, we focused on a CCT program focused on training auditory processing, as we felt it met the needs of these patients. Verbal learning and memory are among the commonly reported cognitive domains to be affected in studies of cognitive outcomes in hematological cancer [30] and may be less amenable to recovery over time as compared to other domains [28, 31, 32]. Consistent with our clinical and research experience, patients treated for hematological cancer also frequently describe difficulty with recalling verbal information (e.g., names, stories, details of conversations), which have been shown to have implications on everyday social and role functioning [33, 34]. Taken together, these findings suggested that a training intervention that targeted the processes supporting the acquisition and processing of auditory information may help meet a clinical need in this population.

## Methods

### Study aims

The primary objective of this study was to evaluate the feasibility of an 8-week home-based, online CCT intervention among patients who have recently completed treatment for hematological malignancy. Secondary objectives were to (i) evaluate the feasibility of recruiting and retaining post-treatment hematological cancer survivors in a prospective study of an online CCT intervention, and (ii) to describe changes in cognitive functioning over the course of the study period.

### Study design and procedure

This study was a single-arm, non-blinded, feasibility study. Given that our aim was to assess feasibility of the intervention, all participants were provided access to the CCT intervention for a period of 8 weeks. Study visits conducted pre-(T1) and post-(T2) the 8-week intervention period collected data on participants’ performance-based and self-reported cognitive functioning. At T2, participants’ perceptions of the intervention were collected. All study visits were conducted in a private room at the study site by a trained assessor under the supervision of an experienced and licensed PhD clinical neuropsychologist. Participants received a $50 honorarium at each study visit.

### Participants and setting

Participants were recruited in hematological oncology clinics at a tertiary cancer centre from February 2016 to September 2016. Eligible participants were between 1 month and 2 years after completion of treatment, comprised of either (i) primary chemotherapy for lymphoma or (ii) autologous stem cell transplantation for lymphoma or multiple myeloma. Additional inclusion criteria included age ≥ 18 years, in stable medical condition, and English-language fluency. Exclusion criteria were active malignancy requiring chemotherapy, severe and uncontrolled psychiatric illness, and previous cancer and/or cancer treatment involving the central nervous system. Individuals with severe hearing impairment were excluded, due to the auditory format of the intervention. Written informed consent was obtained from all participants. The study was approved by the Research Ethics Boards of the University Health Network and the University of Toronto.

### Intervention

Participants were registered for individual accounts to an online CCT program (Auditory Intensive program from Brain HQ^TM^ by Posit Science [26]) that could be administered using their own computer. The program comprises a series of six exercises designed to improve the speed and accuracy of auditory processing information, by presenting auditory stimuli in gradually increasing amplitude and complexity adjusted based on the user’s individual performance [18]. This program has been associated with improvements in higher-order processing-related tasks, as well as measures in non-trained domains, such as memory and attention, and perceptions of cognitive ability [35].

Participants were instructed to complete 40 sessions (1 h/day, 5 days/week) over the 8-week intervention period [35–37]. Login frequency and session duration were tracked by the CCT software. Prior to commencing the program, participants received instructions on how to navigate through the online training exercises and intervention tracking logs. The CCT was self-administered and unsupervised. To address any technical issues, participants were contacted weekly by phone and a study contact number was provided.

### Feasibility of CCT in the early post-treatment period

Our assessment of feasibility focused on participant adherence to the intervention dose and participants’ perceptions.

Adherence was defined as completion of ≥ 30 h of the intervention [18]. We calculated hours of program usage through participant-recorded daily logs, supplemented with data from the CCT tracking system.

Participant perceptions were assessed through an acceptability questionnaire and a semi-structured interview at follow-up. The questionnaire asked patients to rate the following: “The program was easy to follow” (strongly disagree/disagree/neutral/agree/ strongly agree) and “How well was I prepared for how to complete the exercises” (not at all prepared/slightly prepared/moderately prepared/ well-prepared/extremely prepared). The semi-structured interviews were conducted using a descriptive qualitative approach [38]. Questions were developed by the research team with the aim of eliciting an understanding of participants experiences in participating in the intervention, perceived barriers in completing the intervention, and suggestions for refinement of the intervention and/or study processes (Table 1). Probing questions were used, as needed, to gain further clarification. Interviews were audio-recorded and transcribed verbatim.

**Table 1:** Sample interview questions

1. What did you find most surprising about the program? 2. What did you like most about the program? 3. What did you like the least about the program? 4. Were any of the parts of the program difficult to use? 5. Did you experieince any barriers to participating in the program? 6. What improvements would you suggest? 7. What would have been helpful to know before starting the program? 8. Who do you think would benefit most from using this program? 9. Please provide any other comments you think would be helpful for us to understand if this program would be useful to patients after treatment?	

### Recruitment and retention

Recruitment rate was defined as the proportion of approached patients that provide consent and are enrolled to the study, and retention was the proportion of enrolled participants who completed the study.

### Cognitive assessment

Performance-based cognitive functioning was assessed using a neuropsychological battery designed to measure functioning in the most affected domains in cancer patients, as per recommendations of the International Cognition and Cancer Task Force [39]. The battery comprised 14 tests across 5 domains (Table 3): (1) *Learning Efficiency/Memory*, (2) *Information Processing/Psychomotor Efficiency*, (3) *Working Memory*, (4) *Executive Functioning*, and (5) *Language*. Raw scores on performance-based cognitive functioning outcome measures were converted to *T*-scores (mean 50, SD 10) based on norms adjusted for age, sex, and education, where applicable. Composite *T*-scores for each domain were calculated as the average of constituent tests in each domain. Overall cognitive impairment was defined by having at least two tests with a score of ≥ 1.5 SD below the mean (*T*-score ≤ 35) or one test with a score of ≥ 2 SD below the mean (*T*-score ≤ 30) [39].

Self-reported cognitive functioning was assessed using the 33-item Patient’s Assessment of Own Functioning Inventory (PAOFI) [45], measuring participants’ perceptions of daily functioning related to memory, language/communication, sensory-perceptual/motor skills, and higher-level cognitive/intellectual functions. Frequency of difficulty experienced on everyday cognitive tasks is rated from “almost never” (0) to “almost always” (5). Total score ranges from 0 to 165, with lower scores indicating better perceived cognitive functioning. The PAOFI has good reliability and validity for measuring perceived cognitive functioning in the cancer setting [46, 47].

### Sample size

As the primary aim was to assess the feasibility and acceptability of the intervention, a formal sample size calculation was not performed. The sample size was based on available resources (i.e., funding, personnel) and appraisal of the information power of the data in relation to the study aims [48].

### Data analysis

We used descriptive statistics to characterize the demographic and clinical variables, study participation variables, and responses to acceptability ratings. Categorical variables were summarized with counts and percentages. Continuous variables were summarized with median and range.

Qualitative interview data were analyzed using thematic analysis [49], as commonly used in qualitative description [50, 51]. Two of the authors (SJM, RV) independently read and re-read the interview transcripts, generated and assigned initial codes to individual data segments, and collated the initial codes into potential themes, considering both the content (i.e., what was said) and the context of the data (e.g., age, employment status) [49, 51]. Meetings were held regularly throughout the process to ensure inter-coder consistency, discuss emerging themes identified individually and compare extracts of data coded within each candidate theme. The development of themes focused on whether the data captured something important in relation to the research question (i.e., intervention acceptability) rather than the frequency of codes, so as to gain a richer understanding of the factors impacting participants’ engagement with the intervention [51]. When the majority of the interviews had been completed, themes and sub-themes were then developed, reviewed against the entire data set, and further refined in an iterative process as additional transcripts became available. Once data saturation had been reached, the final themes were then validated in discussion with the research team.

Rigor of data analysis was addressed as follows. Credibility of the data was enhanced by the use of investigator triangulation in initial coding of the transcript data, debriefing with the research team, and presentation of results using illustrative quotes. Transferability was ensured through the detailed reporting of the context in which the interview data were elicited. Dependability (reliability) and confirmability was enhanced by investigator triangulation in the analytic process and the maintenance of an “audit trail,” comprised of raw interview transcripts, and drafts of initial codes, notes regarding analytic decisions, and themes [52].

Differences in cognitive functioning outcomes between T1 and T2 were reported using confidence intervals. Statistical analyses were performed using SAS version 9.4 of the SAS system for Windows (©2002–2012 SAS Institute, Inc., Cary, NC).

## Results

### Participant characteristics

**Fig. 1:**
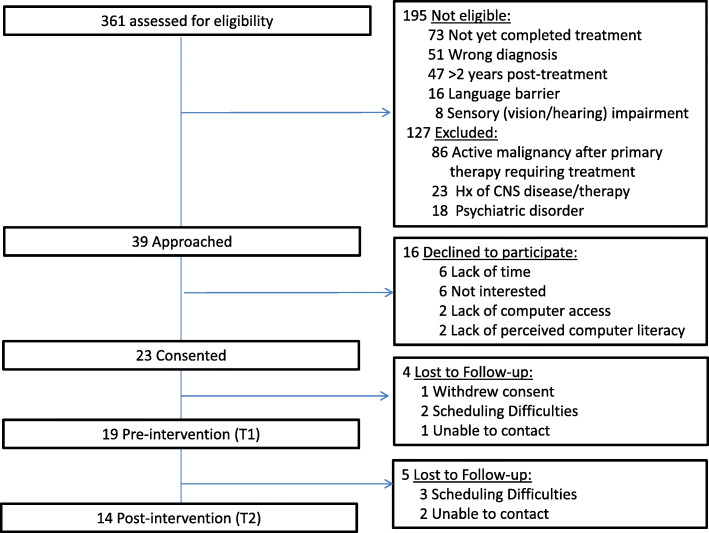
Flow diagram of study participants

Nineteen participants were enrolled in the study (Fig.1 and Table 2). Over half of the participants were male (*n* = 11, 58%), with a sample median age of 52 years (range 22–70). Most participants (84%) had a diagnosis of lymphoma. The median time from completion of treatment to study enrollment was 59 days (range 28–490).

**Table 2:** Sample characteristics

	*N* = 19
Age, median (range)	52 (22–70)
Sex, *n* (%)	
Male	11 (58%)
Female	8 (42%)
Race/ethnicity, *n* (%)	
Caucasian	14 (74%)
Asian	2 (11%)
Black or African American	1 (5%)
Other/not reported	2 (11%)
Education, *n* (%)	
High school diploma	3 (16%)
Some college/university	7 (37%)
Bachelor’s degree	8 (42%)
Postgraduate degree	1 (5%)
Diagnosis, *n* (%)	
Hodgkin lymphoma	10 (53%)
Non-Hodgkin lymphoma^a^	6 (32%)
Multiple myeloma	3 (16%)
Treatment, *n* (%)	
Primary treatment^b^	9 (47%)
Autologous stem cell transplant^c^	10 (53%)
Days post-treatment, median (range)	59 (28–490)

Percentages may not add to 100% due to rounding

*HL* Hodgkin lymphoma, *MM* multiple myeloma, *NHL* non-Hodgkin lymphoma

^a^High-grade NHL (*n* = 5); indolent NHL (*n* = 1)

^b^HL (*n* = 4); high-grade NHL (*n* = 4); indolent NHL (*n* = 1)

^c^HL (*n* = 6); high-grade NHL (*n* = 1); MM (*n* = 3)

### Participant adherence

**Fig. 2:**
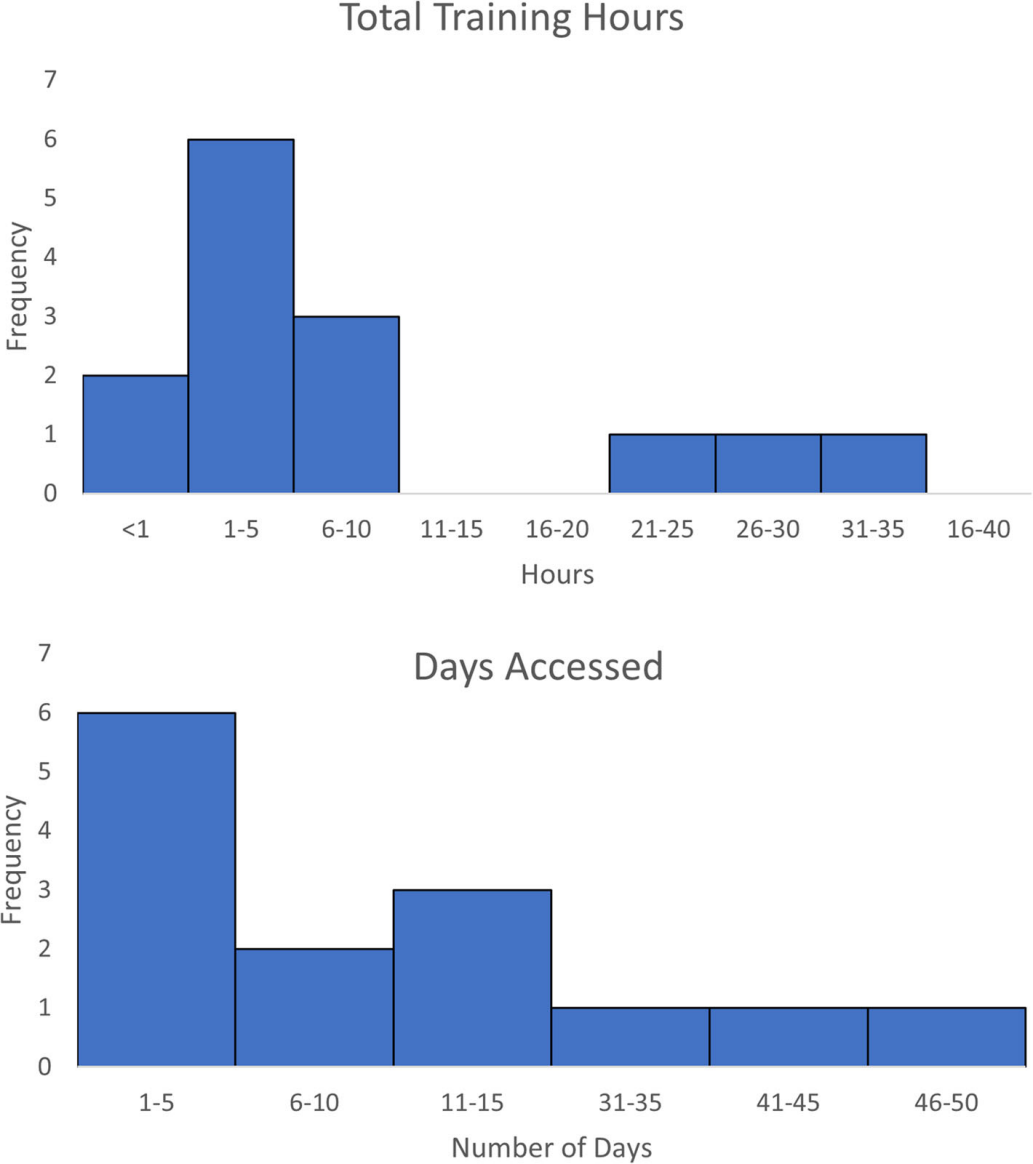
Training activity of participants (*n* = 14)

Median intervention usage for the 14 participants that completed the study was 2.76 h (range 0.0–32.96); one participant met the adherence threshold of 30 h (Fig. 2). No technical difficulties restricting program usage were reported. In a post hoc analysis, daily usage of the program was examined. Participants accessed the online program a median of 6 days during the intervention period (range 1–46), for a median 0.66 h (range 0.26–1.38 h), or 39.6 min, per day.

### Participant perceptions

Participants characterized the program as easy to follow (92%) and felt well-prepared for how to complete the exercises (100%). The length of interviews ranged from 5 to 26 min, with an average length of 13.46 min (SD 6.02). Common themes were generated from the interviews related to barriers to adherence and suggestions for intervention refinements.

#### Barriers to adherence

Participants identified barriers related to (i) interference of physical symptoms and (ii) competing time demands. Changes in physical well-being (mostly associated with fatigue in this transitioning period) impacted some respondents’ ability to focus on cognitive tasks for an extensive period of time and also their willingness to continue engagement in the intervention. As one participant stated:

I would totally do this and I think a lot of other patients would totally do this, too, but they can’t be bothered right now [..…] It’s a hassle to do anything and everything when you’re sick. I don’t want to do it. Like some days you say ‘I really got to do this’ and then you really ponder it and you’re like, ‘do I really need to do it?’ or go back to sleep. (ID:4)

In addition, participants who were negotiating their return to work or school described challenges with managing persistent effects of treatment (e.g., fatigue) and possible changing roles as a consequence of their diagnosis. The context of these participant’s lives meant that many competing priorities influenced the patient’s ability to engage in CCT regardless of their intention to do so. Even though the program was presented in such a way that there was flexibility on when the tasks could be completed, participants were recommended to complete 1 h of intervention per day and found it challenging to meet this expectation:

There’s always something going on in your life and to just dedicate an hour, no distractions and to do this it’s just not going to happen. (…) It’s hard to focus. It’s hard to… it’s hard to fit it in. (ID:1)

#### Suggested intervention refinements

Participants proposed supports that could facilitate adherence to the intervention: (1) formal accountability, (2) personalized feedback and coaching, and (3) peer support. Participants suggested more formal accountability over their participation either by a more rigid scheduling structure for the intervention (e.g., having specific assigned days for task completion) or receiving daily reminders preferably by phone. For those who completed more hours of the program, consistency depended on establishing the task as part of daily activities and making a conscious decision to adhere regularly to the task:

I have to just finish that one session before I can do my other stuff that day. …it’s like anything else once you know you have to do it, the only way to get it done is to do it. Finding your own way around your self-motivation (ID:12)

For others, particularly given the context of barriers to adherence, they recommended that providing greater structure could assist them in maintaining a routine:

If you had a schedule…made a schedule for the person and said like hey so in this and this time period when do you think you are able to work (on the program). (ID:8)

In addition, receiving personalized feedback and coaching on their performance was described as something that could have fostered motivation through a sense of achievement. Within the training program, participants earned “stars” with improved performance and how well participants perceived they were doing impacted their overall mood and also their willingness to continue with the training. Perceived improvement imparted a sense of accomplishment that instilled a desire to continue engaging with the cognitive training intervention.

Because you want to have wings as you go. You want to feel like you’re accomplishing something. (ID:14).

However, receiving personalized feedback and coaching was seen as a way that could help them understand their performance in the context of their recovery and provide them with the encouragement to keep going. This seemed to be particularly important in the circumstance in which participants perceived a lack of progress on the tasks, which contributed to feelings of dejection causing participants to stop the tasks earlier than they anticipated and willingness to return to the intervention thereafter. Having personalized feedback and coaching was viewed as a way to contextualize achievements and help maintain motivation.

Whereas for someone who’s trying to recover from something the first thing you want to do is start feeling better about yourself...You want to feel like you’re accomplishing something. And you come to terms with…it’s going to take a bit of time for you to get better and recover. If (the program) is happening too fast.., I just got frustrated and felt like, I know I’m impaired and I felt like this thing was telling me in my face, hey you’re not as good as all these other people. (ID:15)

If we had (someone) beside us every single day – ‘are you ready for this’, ‘Let’s do this!’ - I’m pretty sure they’d do it 100%. (ID:4)

Participants also suggested the inclusion of peer support through the online program could have also facilitated greater engagement in the program. This was described as allowing for shared experience and opportunity to support one another. Patients thought that engaging with others in the same situation (cancer patients completing the intervention) would be helpful not only to share common grievances and difficulties but to uncover potential solutions to problems and motivate each other on the road to recovery.

…like a little forum where people can say ‘I felt this was helpful to do it at this time of day’ … where people can discuss what they didn’t understand and help someone explain it to them because sometimes it is easier to talk to another patient or person doing this than the person who is supervising you. (ID:3)

However, participants also acknowledged that individuals’ willingness to engage in group communication could vary widely, and the level of involvement with peers should be something that individuals can control. One participant described the potential risks of being in contact with a group, particularly during an intervention that might challenge one’s ability:

…could be intimidating for others, (since) they know that they don’t want to look inferior (ID:13)

### Recruitment and retention

In total, 39 patients were approached, 23 of whom consented to participate in the study (*recruitment rate* 59%). Documented reasons for refusal were lack of interest in the intervention (*n* = 6), lack of time (*n* = 6), and lack of computer access (*n* = 4). After providing consent, one participant withdrew because of lack of interest and three were lost-to-follow-up. Of the 19 participants enrolled in the study, 14 completed T1 and T2 assessments (*retention rate* 74%). The flow diagram of study participants is provided in Fig. 1.

### Change in cognitive outcomes

Performance in the domains of information processing speed/psychomotor efficiency (MD = 4.18, 95% CI 1.55–6.81) and working memory (MD = 2.09, 95% CI 0.5 1–3.67) improved between T1 and T2 (Table 3). No differences were observed in learning efficiency/memory, executive functioning, or self-reported cognitive functioning. Eleven participants (58%) and eight participants (57%) met criteria for overall cognitive impairment at T1 and T2, respectively.

**Table 3:** Cognitive outcomes at T1 and T2

	T1 (*n* = 19)		T2 (*n* = 14)		MD^a^ (95% CI)	Std
	Median	Range	Median	Range		
Learning efficiency/memory	42.00	24.00–57.50	38.75	20.00–62.50	− 0.36 (− 6.35–5.94)	10.39
HVLT-R total recall	40	20–56	38.5	20–62		
HVLT-R delayed recall	40	27–61	41	20–63		
Information processing/psychomotor efficiency	44.50	28.50–54.80	45.63	29.25–63.25	4.18 (1.55–6.81)	4.55
TMT-A	45	28–67	51.5	32–64		
WAIS-III digit symbol	46	30–63	47	30–70		
Grooved Pegboard—dominant	39	11–58	42	27–63		
Grooved Pegboard—non-dominant	37	22–57	41	20–58		
Working memory	49.25	37.00–59.30	52.75	41.75–57.00	2.09 (0.51–3.67)	2.74
TMT-B	43	24–63	48.5	34–59		
WAIS-III letter number sequencing	50	37–67	50	37–73		
WAIS-III digit span	48.50	33–63	53	40–67		
WMS-III spatial span	53	40–63	50	37–63		
Executive functioning ^b^	45.33	36.33–66.7	48.33	33.33 – 63.00	− 0.11(− 5.93–5.70)	7.57
WCST—total errors	48	31–80	52	29–69		
WCST—perseverative responses	46.50	37–80	51	38–80		
WCST—categories	40	37–40	40	33–40		
Language (FAS)	38.50	25.00–55.00	40.50	25.00–55.00	0.79 (− 2.67–4.24)	5.99
Impaired, *n* (%)	11 (58%)		8 (57%)			
Patient’s assessment of own functioning	22.84	1.00–86.00	23.69	2.00–55.00	− 3.92(− 11.14–3.30)	11.95

*HVLT-R* Hopkins Verbal Learning Test-Revised [40], *TMT-A* Trail Making Test-Part A [41], *TMT-B* Trail Making Test-Part B [41], *WAIS-III* Wechsler Adult Intelligence Scale-III [42], *WMS-III* Wechsler Memory Scale –III [43], *WCST* Wisconsin Card Sorting Test [44]

^a^Pairs used = 14, except for Executive Functioning domain where pairs used = 9

^b^*n* = 16 at T1, *n* = 9 at T2

## Discussion

To date, the majority of studies focused on home-based CCT interventions in cancer have focused on long-term survivors of solid tumors. This study investigated the feasibility and acceptability of an unsupervised, home-based, online CCT intervention after treatment for hematological malignancy, focusing on the first 2 years after treatment completion. As assessed through an a priori threshold of 30 total hours of intervention usage, we were unable to demonstrate feasibility of the intervention as designed. However, qualitative interviews highlighted strengths of the intervention, challenges that contributed to limited adherence, and potential improvements that could enhance the acceptability of CCT interventions in this setting.

The early post-treatment phase has been identified as a period of transition that can be challenging for patients to manage, but a time in which supportive care is most needed [53]. To optimize access and flexibility, the CCT intervention was administered in a home-based, online format. Overall, patients reported that the exercises were easy to understand and navigate through without supervision, and some participants found the exercises enjoyable. However, participants described major barriers to intervention adherence were concurrent symptoms and competing demands related to re-integration. In this context, maintaining one’s motivation was viewed as necessary to facilitating adherence to the CCT program, and participants suggested improvements including the introduction of more formalized accountability, personalized feedback, and peer support. Given that behavioral change is effectively facilitated by interventions that foster self-regulatory skills and promote problem-solving [54], behavioral supports specific to CCT could target how to manage the barriers related to symptoms and time demands. Given the symptom burden and cognitive difficulties among the participants, it is possible that they may also benefit from more frequent training reminders from the study team, as in a previous study [29].

A major factor affecting adherence to the program was the recommended training time. We found that the recommended training dose of 40 h was not achievable in the months immediately after hematological cancer treatment, and additionally discouraged adherence for some participants. Lower training times have been associated with improvements to cognitive outcomes in other cancer settings. Bray et al. [21] conducted a pragmatic RCT of an unsupervised 15-week CCT intervention among 242 patients of mixed cancer diagnoses, a mean 27 months from completion of chemotherapy. The intervention group had an average training time of 25 h and demonstrated improved self-reported cognitive symptoms that were sustained six months later. In a RCT of 41 long-term breast cancer survivors, Kesler et al. [23] demonstrated improvements in both self-reported cognitive problems and objective measures of processing speed and executive function, with a dose of 48 sessions, 20–30 min each (approx. 24 h) over 12 weeks. While comparisons to existing trials are challenging due to heterogeneity in study populations, outcome measures, and training platforms, successful implementation of CCT in the post-treatment period will likely require the determination of an optimal dose that balances feasibility with clinically meaningful effectiveness on cognitive outcomes. In our study, participants completed on average 38 min each session. In light of our qualitative findings, reducing the daily-recommended dose may enhance the feasibility of sustained engagement in the training exercises. To the extent that adherence is used as a criteria for participant inclusion in analyses of intervention efficacy, some studies of cognitive training have used a lower threshold of adherence (e.g., 10 h) while detecting notable cognitive changes [29, 55], suggesting a lower operationalization of adherence may be reasonable.

In this feasibility study, we also sought to evaluate recruitment and study retention. Notwithstanding the need for intervention refinement, our enrollment and retention rates provide tentative support for the feasibility of testing cognitive interventions in this population. Whereas previous cognitive intervention trials have focused on long-term cancer survivors, we were able to demonstrate a willingness of patients to participate in a cognitive intervention study within the first few months of completing treatment, with limited loss-to-follow-up over an 8-week study period. The testing of interventions within this post-treatment phase may lead to effective early interventions to prevent or minimize prolonged impacts of cognitive impairment on quality of life.

Training programs that focus on speed of processing have been shown to improve processing speed but differ in transfer effects to/in other non-trained domains [22, 24, 29]. Another important point to raise is the possibility that while some neurocognitive abilities may improve with training on a shorter time period (e.g., improvements in speed of processing and complex attentional skills and working memory over weeks), other more complex and higher-level neurocognitive abilities (e.g., mnestic functions and executive skills) may require longer and persistent periods of active intervention (over months) to show meaningful changes and/or recovery. There may also be a sequence of changes which occur, such that improvements in more basic neurocognitive functions need to recover or normalize, before more complex neurocognitive functions (that require more complex cognitive networks) can recover. We observed improvements in information processing speed/psychomotor efficiency and working memory performance over the course of the intervention period, but did not observe any changes in other objectively measured domains or participant’s perceptions of their own cognitive functioning. The interpretation of this result as it relates to the intervention would remain speculative in light of the limited intervention adherence in this study. Given documented improvements in cognitive functioning within the acute post-treatment period and potential for improved performance due to increasing familiarity with study measures, the degree to which our findings reflect the natural trajectory of cognitive recovery and/or practice effects remains uncertain. To clarify intervention effects, adequately powered studies with a suitable control group are needed.

We acknowledge the limitations of this study. Our study is based on a small sample of participants who were heterogeneous with respect to diagnostic groups, treatment received, and time from treatment, all of which may have influenced the variability in intervention adherence. We also did not limit enrollment to individuals with cognitive complaints and acknowledge that such individuals may be more motivated to complete CCT exercises. However, with our existing sample, we were able to gain valuable insights regarding important considerations that need to be addressed when testing cognitive rehabilitation interventions in the post-treatment period across a range of hematological cancer patients with varying degrees of cognitive complaints. Notably, over half of the participants met the criteria for cognitive impairment at the start of the intervention period despite low levels of cognitive complaints as measured on the subjective questionnaire, suggesting a potential need for testing interventions that impact cognitive impairments that may not be perceived by patients themselves. Our findings suggest that CCT participation in the post-treatment period may be challenging. Further research on the effectiveness of CCT for CRCI in this setting may require the implementation of strategies that help sustain motivation over time and help balance the intervention with competing concerns, including establishing a minimum dose requirement.

## Conclusion

As mechanisms underlying the development of CRCI remain speculative [56, 57], there is an impetus for the development of non-pharmacological interventions to alleviate CRCI, such as CCT. Overall, our findings suggest that CCT participation in the post-treatment period may be challenging. We were unable to demonstrate the feasibility of a 40-h dose of unsupervised, home-based, online CCT in post-treatment hematological cancer survivors, but were able to gain valuable insights into potential factors affecting CCT adherence. Future trials of CCT in this population should build on our qualitative findings when considering intervention design and leverage findings from studies that have applied a lower dose and/or threshold for adherence.

## Data Availability

The datasets during and/or analyzed during the current study available from the corresponding author on reasonable request. Portions of this research were presented as poster presentations at the MASCC/ISOO Annual Meeting on Supportive Care in Cancer, June 22–24, 2017, Washington DC, USA; and 59^th^ American Society of Hematology Annual Meeting and Exposition, December 9–12, 2017, Atlanta, GA, USA.
